# Case Report: Mixed ductal–lobular carcinoma consisting of invasive lobular carcinoma with a glycogen-rich clear cell pattern and elevated tumor mutation burden

**DOI:** 10.3389/fonc.2026.1741727

**Published:** 2026-01-26

**Authors:** Kae Kawachi, Xiaoyan Tang, Rika Kasajima, Kotoe Katayama, Rui Yamaguchi, Kiyoshi Yamaguchi, Yoichi Furukawa, Seiya Imoto, Satoru Miyano, Emi Yoshioka, Kota Washimi, Yoichiro Okubo, Shinya Sato, Tomoyuki Yokose, Yohei Miyagi

**Affiliations:** 1 Department of Pathology, Kanagawa Cancer Center, Yokohama, Japan; 2 Department of Pathology, The Jikei University School of Medicine, Tokyo, Japan; 3 Department of Pathology, Nihon University Hospital, Tokyo, Japan; 4 Molecular Pathology and Genetics Division, Kanagawa Cancer Center Research Institute, Yokohama, Japan; 5 Division of Health Medical Intelligence, Human Genome Center, Institute of Medical Science, The University of Tokyo, Tokyo, Japan; 6 Division of Cancer Systems Biology, Aichi Cancer Center Research Institute, Nagoya, Japan; 7 Division of Cancer Informatics, Nagoya University Graduate School of Medicine, Nagoya, Japan; 8 Division of Clinical Genome Research, Institute of Medical Science, The University of Tokyo, Tokyo, Japan; 9 M&D Data Science Center, IIR, Institute of Science Tokyo, Bunkyo, Japan; 10 Department of Pathology, Odawara Municipal Hospital, Odawara, Japan

**Keywords:** breast cancer, glycogen-rich clear cell pattern, invasive lobular carcinoma, mixed ductal-lobular carcinoma of the breast, phylogenetic analysis, SETD2

## Abstract

**Background:**

Mixed ductal–lobular carcinoma (MDL) of the breast exhibits considerable molecular complexity. The pathways leading to the glycogen-rich clear cell morphology of the breast tumors, and its clinical relevance, currently remain unclear. Herein, we report a case of MDL, predominantly composed of invasive lobular carcinoma with a glycogen-rich clear cell pattern (gILC), accompanied by classic invasive lobular carcinoma and invasive ductal carcinoma (IDC).

**Case presentation:**

A 70-year-old woman presented with a 3.5 cm mass in the left breast, for which total mastectomy was performed. The pathological diagnosis was MDL predominantly comprising gILC. Tissue samples from the gILC and IDC areas were subjected to whole-exome and RNA sequencing. The gILC region had a higher tumor mutation burden than the IDC. Three stop-gain single nucleotide variations (SNVs) in *CDH1*, *SETD2*, and *USP9* and two nonsynonymous SNVs in *PIK3CA* were identified in the gILC region, whereas only two nonsynonymous SNVs in *SMAD4* and *PIK3CA* were identified in the IDC region. Phylogenetic analysis revealed a common ancestor of gILC and IDC, sharing a pathogenic *PIK3CA* p.H1047L mutation. Reduced SETD2 protein and H3K36me3 levels and the DNA mismatch repair-microsatellite instability-associated mutational signatures SBS6 and SBS26 were uniquely demonstrated in gILC. Further, a structural variant involving *HNF1B* and elevated *HNF1B* transcript levels was detected in gILC. The predominant gILC component was estrogen receptor-positive. Adjuvant endocrine therapy was administered postoperatively, and the patient currently remains disease-free at 51 months.

**Conclusion:**

In this case, the gILC and IDC components of an MDL shared a common origin, but exhibited marked genomic divergence. This experience also shows that SETD2 functional impairment may underlie gILC hypermutation, while HNF1B overexpression could contributes to a glycogen-rich clear cytoplasm. Overall, this case emphasizes the complexity of MDL with gILC, and highlights the need for further studies to clarify the underlying molecular mechanisms and their prognostic implications.

## Introduction

The glycogen-rich clear cell pattern is a rare morphological pattern of invasive breast carcinoma of no special type (IBC-NST), also referred to as invasive ductal carcinoma (IDC). This pattern is characterized by an abundant clear cytoplasm containing glycogen (1). Fisher et al. first described 45 cases of glycogen-rich clear cell breast cancer in 1985, characterizing this condition by the presence of tumors with > 50% tumor cells containing an optically clear cytoplasm, often with a central nucleus (2). Glycogen-rich clear cells appear as sheet-like, nested, or corded growth patterns, but they also display papillary, tubular, or lobular patterns (1). Differential diagnoses include lipid-rich, sebaceous, and histiocytoid patterns of IBC-NST/IDC; secretory carcinoma; myoepithelial tumors; and, although rare, metastatic tumors, such as clear cell renal cell carcinoma (ccRCC). The prognosis of carcinomas with a glycogen-rich clear cell pattern is still debated owing to their rarity (3, 4). The glycogen-rich clear cell pattern is rare in invasive lobular carcinoma (ILC), and its impact on tumor behavior remains unclear.

ILC is the most common special type, accounting for 5–15% of invasive breast carcinomas. In total, 5% of invasive breast carcinomas contain both ductal and lobular morphologies (5), which are categorized as “mixed IBC-NST/IDC and ILC (mixed ductal–lobular carcinoma [MDL])” if the ILC comprises 10–90% of the tumor. Although some MDLs may be collision tumors, several studies have demonstrated that the ductal and lobular components of MDLs are clonal and originate from the same ancestor, and lobular morphology can arise via a ductal pathway (6–9). These invasive carcinomas may acquire the lobular phenotype through E-cadherin inactivation (6). The mechanism underlying this phenotypic change seems to involve the dysregulation of the E-cadherin–catenin complex. However, the precise molecular mechanisms have not been fully elucidated owing to the significant heterogeneity among these tumors.

We reported a case of MDL predominantly composed of an ILC with a glycogen-rich clear cell pattern (gILC), accompanied by a classic ILC without a glycogen-rich clear cell pattern (cILC) and an IDC. This study aimed to determine whether lobular and ductal carcinoma components share a common ancestry and to explore the potential mechanisms underlying gILC. Therefore, we analyzed the genetic mutations and expression profiles of gILC and IDC components.

## Case report

A 70-year-old woman presented with a palpable mass in the left breast. She had no family history of breast or ovarian cancer. Ultrasonography revealed the main mass and two smaller nodules. Core-needle biopsy of the main mass indicated an ILC. Total mastectomy with sentinel lymph node biopsy was performed.

**Figure 1:**
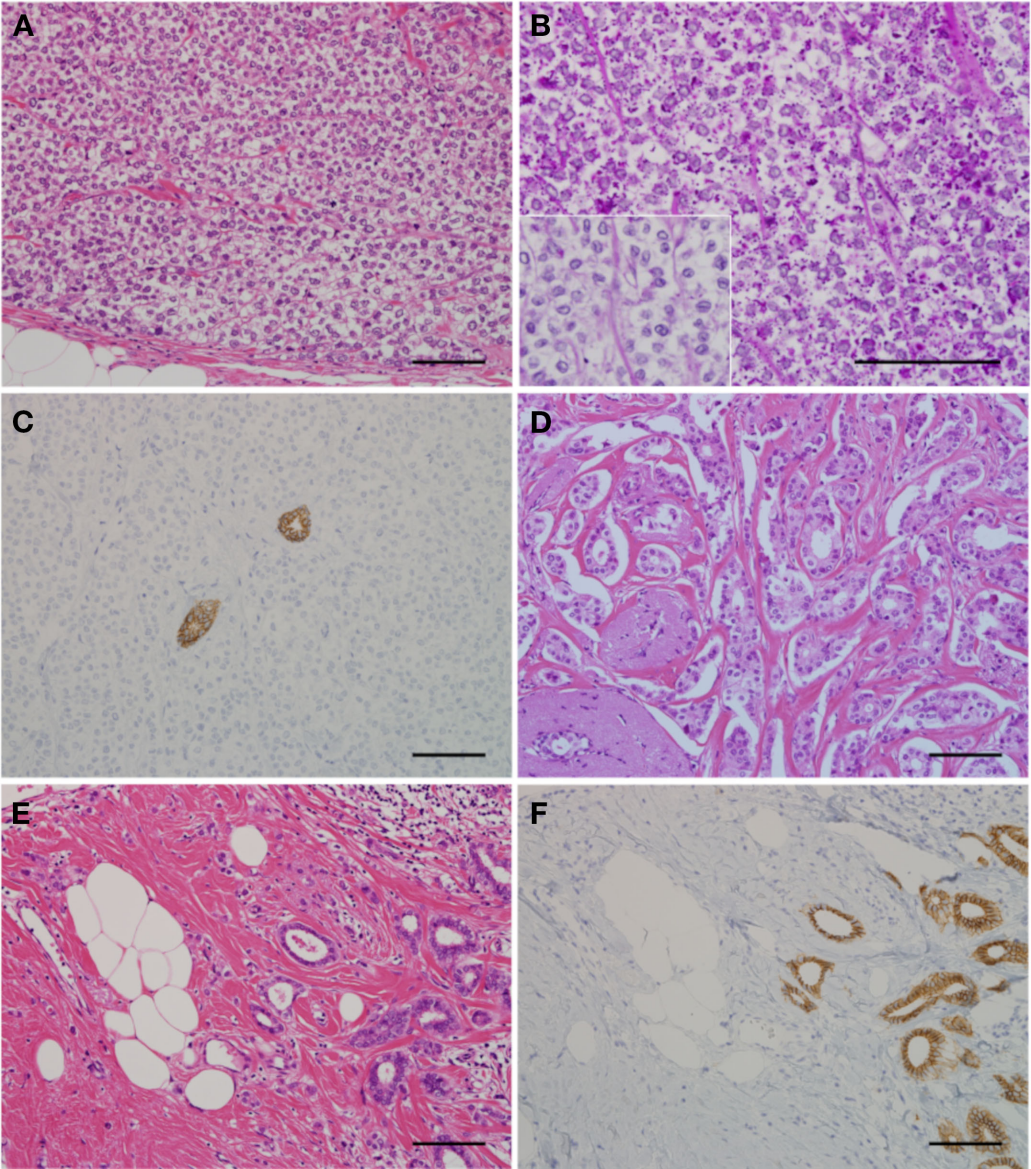
Histological and immunohistochemical features of mixed ductal–lobular carcinoma. **(A)** The gILC component showed diffuse proliferation of invasive carcinoma with clear cytoplasm and apparent cell boundary without gland formation. **(B)** Tumor cell cytoplasm of gILCs were PAS-positive with diastase digestion (inset). **(C)** Immunohistochemical study identified that the gILC tumor cells were E-cadherin-negative. **(D)** IDC component showed ductal, small nested, and trabecular pattern. **(E)** An admixture of cILC and IDC was present in a significantly limited area. **(F)** E-cadherin was negative for the cILC component and positive for the IDC component. Scale bar = 0.1 mm. IDC, invasive ductal carcinoma; gILC, invasive lobular carcinoma with glycogen-rich clear cell pattern.

**Figure 2:**
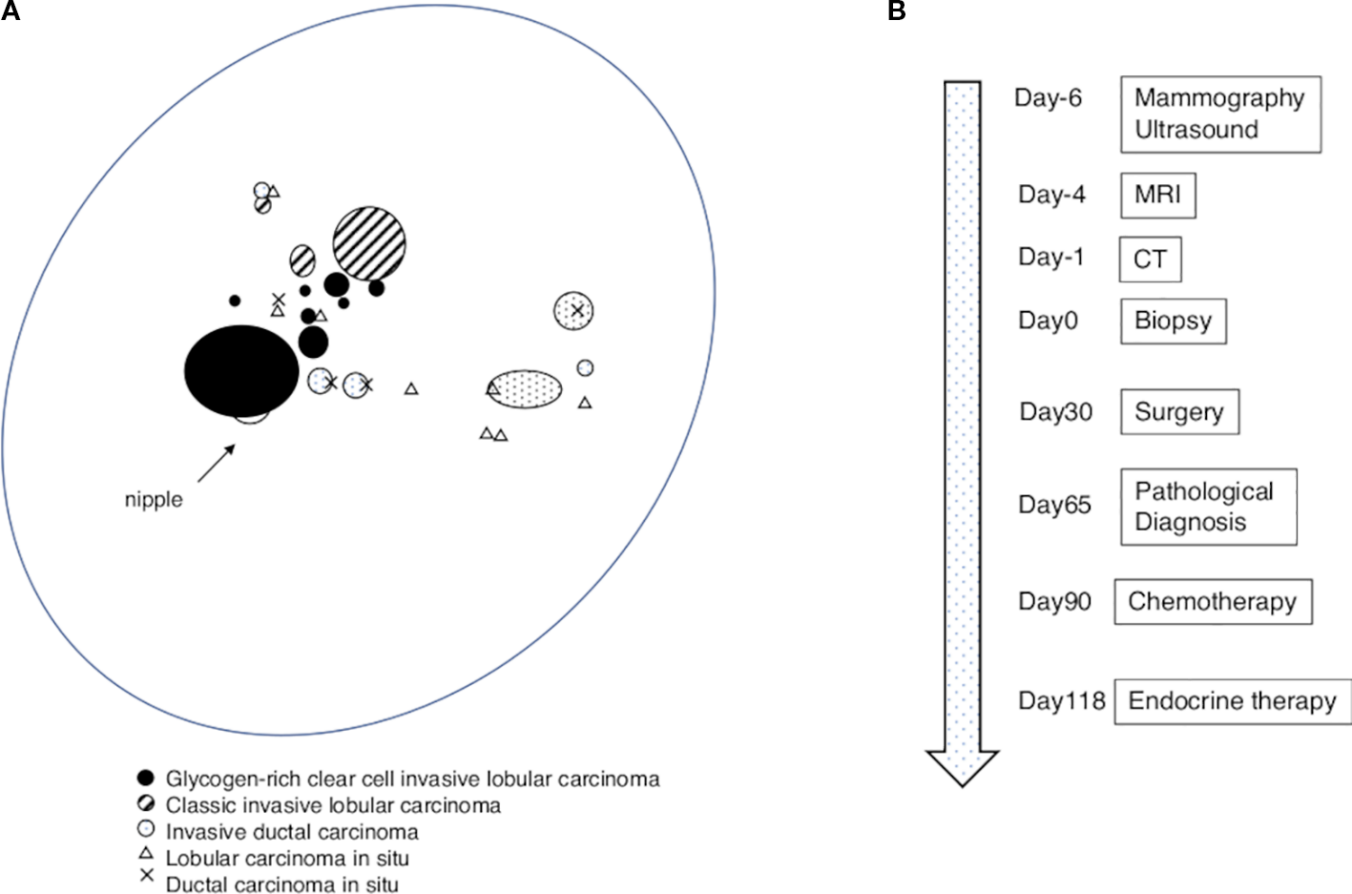
Overview of the lesion and clinical course. **(A)** Schema showing the distribution of different histologies in the left breast. **(B)** Disease timeline aligned to Day 0, defined as the date of core-needle biopsy confirming invasive lobular carcinoma; events are shown relative to Day 0, including imaging studies, biopsy, surgery, chemotherapy and endocrine therapy.

Gross examination of the mastectomy specimen revealed multiple ill-defined masses beneath the nipple, extending to the lateral areas. The largest mass measured 3.5 cm in diameter. Microscopically, the central area of the largest tumor revealed diffuse proliferation of the invasive carcinoma with a clear cytoplasm and an apparent cell boundary without gland formation (**Figure 1A**). Cytological atypia was moderate to focally high, with seven mitoses per 10 high-power fields. The cells containing cytoplasmic dots stained with periodic acid-Schiff were digested by diastase (**Figure 1B**) and were immunohistochemically E-cadherin-negative (**Figure 1C**), suggesting that the tumor was a gILC. Among the invasive lesions, noninvasive lobular carcinoma with a gILC was also observed. A mass consisting of cILC was observed in the upper lateral area. The gILC and cILC components were intermingled within numerous microscopically infiltrating foci located between the tumor masses. In the central to lower lateral area, an IDC with ductal, small nested, and trabecular patterns was observed, including a small number of noninvasive ductal components (**Figure 1D**). The IDC was histological grade I. An admixture of cILC and IDC was observed over a small area (**Figures 1E, F**). Classic noninvasive lobular carcinoma was also found in the background. The final diagnosis was MDL, predominantly composed of gILC. The distribution of different histologies in the left breast is shown schematically in **Figure 2A**. The gILC components were estrogen receptor-positive (> 90% of the cells) and progesterone receptor-negative (< 1% of the cells). The Ki-67 proliferative indices were 30%, 6%, and 10% in the gILC, cILC, and IDC (hot spot) components, respectively (**Supplementary Figure S1**). The HER2 immunohistochemical score of the gILC component was 1 +. Sentinel lymph node biopsy was negative (pN0[sn]). In summary, the final pathologic stage was pT2N0M0.

After surgery, the patient received adjuvant docetaxel and cyclophosphamide, which was discontinued after one cycle owing to adverse effects. The patient subsequently received endocrine therapy and was alive and disease-free at 51-month follow-up (**Figure 2B**).

Genomic DNA and total RNA were extracted separately from formalin-fixed and paraffin-embedded (FFPE) tissue slices of gILC, IDC, and nonneoplastic breast tissue by macrodissection for whole-exome sequencing, targeting protein-coding exons and exon–intron junctions, and RNA sequencing. Detailed methods are provided in **Supplementary Methods**.

**Figure 3:**
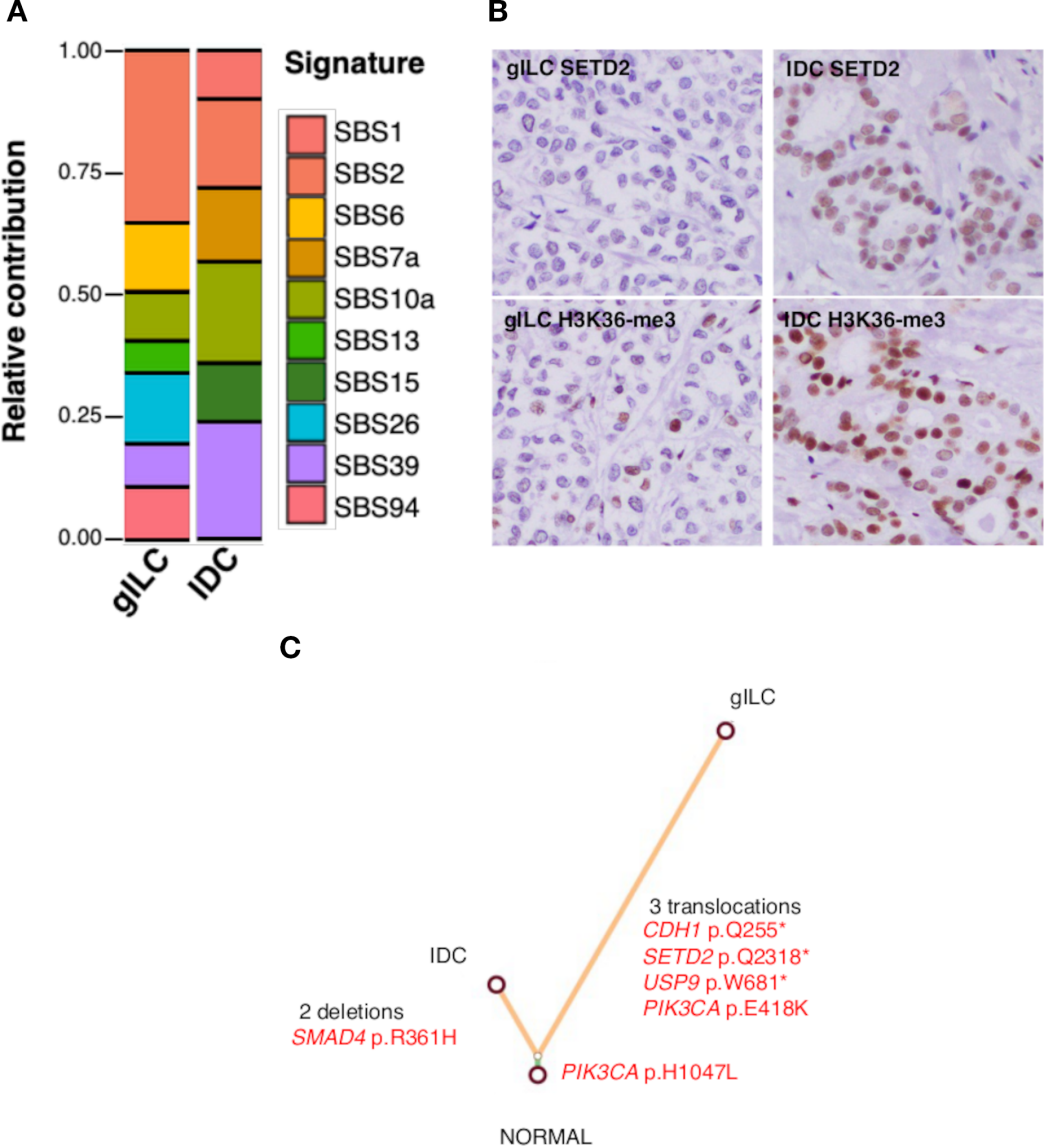
Overview of the genetic alterations in the lobular and ductal regions. **(A)** Analysis of the COSMIC single-base substitution mutational signature in gILC and IDC. The SBS6, 13, 26, and 94 were unique to the gILC, whereas SBS1, 7a and 15 were unique to the IDC. **(B)** Immunohistochemical analysis of SETD2 and H3K36-me3 expressions in gILC and IDC. Representative images show markedly reduced SETD2 expression in gILC cells when evaluated with an antibody recognizing the carboxyl-terminal region. Consistently, the level of H3K36-me3, the histone mark catalyzed by SETD2 methyltransferase activity, is also lower in gILC compared with IDC. **(C)** Clonal evolution analysis on mixed ductal–lobular carcinoma. A phylogenic tree of the tumor using neighbor-joining algorithm was constructed from both components of the gILC and IDC. Somatic single-nucleotide variants and indels from whole-exome data were used. Branches were colored according to the regional distribution of the mutations. The branch lengths are proportional to the number of mutations. The *PIK3CA* p.H1047L mutation was common between IDC and gILC. Only pathogenic mutations were listed. IDC, invasive ductal carcinoma; gILC, invasive lobular carcinoma with glycogen-rich clear cell pattern.

All somatic single nucleotide variations (SNVs), small insertions and deletions (indels), and structural variations (SVs) that met the criteria set are listed in **Supplementary Tables S1**-**S5**. In total, the gILC components harbored 616 SNVs and indels, whereas the IDC components harbored 158. The tumor mutation burden (number of SNVs and indels/Mb sequenced) values were 18.2 for the gILC and 4.7 for the IDC. Three stop-gain SNVs in *CDH1* (p.Q255*), *SETD2* (p.Q2318*), and *USP9* (p.W681*) and two nonsynonymous SNVs in *PIK3CA* (p.E418K, p.H1047L) were identified in the gILC. In contrast, only *SMAD4* (p.R361H) and *PIK3CA* (p.H1047L, identical to that found in gILC), were found in the IDC (**Supplementary Tables S1**, **S2**). Analysis of the COSMIC single-base substitution mutational signature (Human Cancer Signatures version 3.4, https://cancer.sanger.ac.uk/signatures/sbs/) revealed that SBS6, 13, 26, and 94 were unique to gILC, whereas SBS1, 7a, and 15 were unique to IDC (**Figure 3A**, **Supplementary Table S3**). Two deletions and three interchromosomal translocations were identified in the IDC and gILC, respectively. No SVs were considered pathogenic or shared between the two components. A translocation identified in gILC connected a non-genic region on chromosome 12 to exon 7 and the downstream 3′ region of *HNF1B* at a site corresponding to amino acid residues p.Val456 and p.Pro457 (NM_000458) (**Supplementary Table S4**).

Based on the identification of the *SETD2* truncation mutation at the carboxyl terminus (Q2318) uniquely in the gILC, we evaluated the expression of the SETD2 protein and tri-methyl-histone H3 lysine 36 (H3K36-me3) in gILC and IDC by immunohistochemistry. Expression of SETD2, evaluated using a primary antibody recognizing the carboxyl-terminal region, was significantly attenuated in gILC. The amount of H3K36-me3, the product generated by SETD2 methyltransferase activity, was also reduced in gILC (**Figure 3B**).

Changes in copy number were similar between IDC and gILC, with no remarkable amplifications or deletions (**Supplementary Figure S2**). We constructed a clonal phylogenetic tree of the tumors using the identified SNVs and indels. A common trunk was visualized consisting of 29 mutations, including the pathogenic *PIK3CA* p.H1047L mutation, and two branches of 158 and 616 mutations unique to IDC and gILC, respectively, were extended (**Figure 3C**).

**Figure 4:**
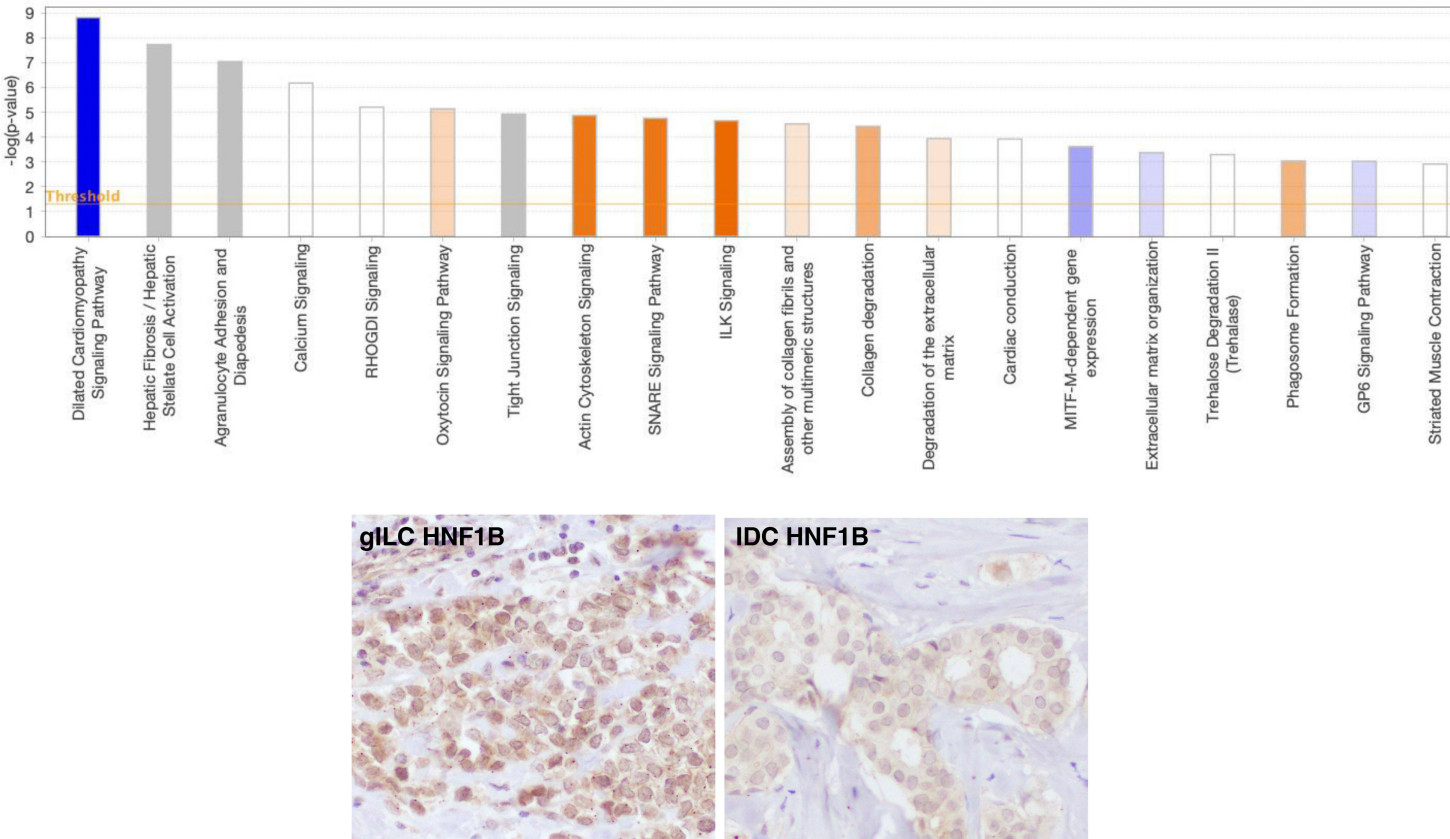
Differentially expressed genes: an enrichment analysis in lobular and ductal regions. **(A)** Canonical pathway analysis of differentially expressed genes between gILC and IDC using Ingenuity Pathway Analysis (IPA). Bar graph show top 20 clusters with representative enriched terms (one per cluster). The height of each bar represents the significance of the enrichment. Orange bars indicate predicted pathway activation (Z-score ≥ 2.0), blue bars indicate predicted pathway inhibition (Z-score ≤ –2.0) in IDC compared with gILC, and gray bars represent pathways where no prediction could be made or with Z-scores between –2.0 and 2.0. The orange line denotes the threshold for significance (p < 0.05). The complete figure showing all data with p < 0.05 is provided in the Additional file 8. **(B)** Immunohistochemical study of HNF1B. HNF1B was diffusely and weakly positive in the nucleus of gILC (left). On the contrary, IDC showed negative result (right). IDC, invasive ductal carcinoma; gILC, invasive lobular carcinoma with glycogen-rich clear cell pattern.

RNA-sequencing analysis identified 288 differentially expressed genes (DEGs) in IDC relative to the gILC components (|log2 fold change| > 2.0, p < 0.05) (**Supplementary Table S6**). Enrichment analysis using Metascape showed that the DEGs were concentrated in pathways, such as “extracellular matrix organization (Reactome Gene Sets)” and “actin filament-based movements (Gene Ontology Biological Processes)” (**Supplementary Figure S3A**). Ingenuity Pathway Analysis revealed significant enrichment of several canonical pathways (**Figure 4A**, **Supplementary Figure S3B**). The “dilated cardiomyopathy signaling pathway” (–log p-value = 8.83, Z-score = –2.828) was the most significantly enriched and was predicted to be significantly inhibited in the IDC. Conversely, “actin cytoskeleton signaling” (Z-score = 2.236), “SNARE signaling pathway” (Z-score = 2.236), and “ILK signaling” (Z-score = 2.449) were predicted to be significantly inhibited in the gILC. “Extracellular matrix organization” was predicted to be activated in the gILC; however, z-score did not reach statistical significance.

Despite the genomic translocation involving exon 7 of *HNF1B*, RNA sequencing detected only the wild-type transcript, with no evidence of a corresponding fusion transcript. However, the expression level of the wild-type *HNF1B* transcript was higher in gILC than in IDC. Immunohistochemistry was performed to comparatively evaluate HNF1B protein expression. Using a primary antibody recognizing a sequence within the central region of human HNF1B, weak expression was observed in gILC, whereas no expression was detected in IDC (**Figure 4B**). However, among the major HNF1B target genes reported by Chandra et al., *GLUT2*, which is involved in glucose metabolism, was not identified as a DEG in the present study (**Supplementary Table S7**) (10).

## Discussion

Here, we reported a case of MDL predominantly exhibiting gILC coexisting with cILC and IDC. Genetic analysis suggested a common cellular origin for the gILC and IDC components that share a pathogenic *PIK3CA* mutation. Only gILC was elevated in TMB, with mutational signatures associated with defective DNA mismatch repair and microsatellite instability (MSI). This hypermutated phenotype appeared to be partly caused by *SETD2* inactivation. The gILC in this case exhibited a solid pattern and higher *HNF1B* expression than IDC.

Glycogens and lipids that accumulate in the cytoplasm are eluted during FFPE tissue processing, causing the cytoplasm to appear clear; however, the mechanism of this accumulation in breast cancer remains unknown. HNF1B is a transcription factor that regulates genes involved in the cell cycle, apoptosis, and oxidative stress responses (11). Cuff et al. found significantly higher HNF1B protein expression in cancer cells with cytoplasmic clearing histology, observed in ovarian clear cell carcinoma and ccRCC, associated with *HNF1B* promoter hypomethylation (12). Bioinformatics analysis revealed that the HNF1B target gene set was rich in genes related to glycogen metabolism, such as glucose-6-phophatase, potentially explaining cytoplasmic clearing due to glycogen accumulation (12). This suggests that high *HNF1B* expression may be involved in the glycogen-rich clear cytoplasm observed in our gILC; however, no evidence of upregulated expression in gILC was observed for glucose metabolism-related genes among the major HNF1B target genes. Although *HNF1B* was disrupted at protein-coding exon 7 by a translocation, levels of the wild-type *HNF1B* transcript, as well as the corresponding protein, were increased in gILC compared with IDC. Given that the disrupted allele frequency was approximately 0.1, the retained wild-type alleles appear to be actively transcribed, although the underlying mechanism remains unclear.Because of its rarity, the mutational profile of gILC has not been differentially analyzed. In contrast, genetic analyses of nine breast cancers with pure clear cell morphology, including six cases with glycogen-rich patterns, have been reported, although their histological subtypes (ductal or lobular) have not been clearly specified (13). This prior study reported mutations in *TP53*, *BRCA2*, *PIK3R1*, *BCOR*, and *PTEN*. None of these were shared with the gILC component of the present case. However, in the context of perturbation of the PI3K signaling pathway, the *PIK3CA* mutation we identified was functionally analogous to *PIK3R1* and *PTEN* mutations, which are frequently observed in breast cancers. In addition, *CDH1* mutation, frequently detected in ILC (37–64%) (14) and associated with its histologically diffusely invasive phenotype, was identified in our gILC case. The genetic profile of our gILC appeared to be broadly similar to that of conventional ILC.

The clonal phylogenetic tree visualized based on the SNVs and indels observed in gILC and IDC revealed a common ancestor of these tumor cells, sharing the pathogenic *PIK3CA* p.H1047L mutation. As suggested by a prior study (6), the presence of both ductal carcinoma *in situ* (DCIS) and lobular carcinoma *in situ* (LCIS) in our case suggests that clonal evolution from DCIS to LCIS may have been preceded by the development of invasive tumors. Alternatively, we could speculate the existence of a further common lesion preceding the development of DCIS. The observation of both glycogen-rich clear LCIS and classic non-clear LCIS indicated that a glycogen-rich clear cell phenotype was obtained at the LCIS stage, which subsequently formed invasive lesions. However, mutational or gene expression data from DCIS, classic non-clear LCIS, LCIS with clear cell patterns, and cILC are required to clarify these findings.

An *SETD2* mutation was identified exclusively in gILC, with a high variant allele frequency of 0.536. SETD2 is the sole H3K36-specific trimethyltransferase that functions in transcriptional elongation, DNA damage repair, and RNA splicing (15). The incidence rates of *SETD2* mutations are 2.6% in invasive breast cancer and 1.2% in triple-negative breast cancer, based on data from TCGA and the METABRIC breast cancer study (16). Further details of the histological subtypes have not yet been specified. The identified truncating mutation p.Q2325* completely abolishes the carboxy-terminal SRI domain (residues 2422–2564), a domain that mediates interaction with the RNA polymerase II CTD and plays a pivotal role in enabling H3K36 trimethylation during transcriptional elongation. *SETD2*-inactivating mutations and decreased H3K36-me3 levels are often found in various types of cancers, indicating tumor suppressor characteristics of *SETD2* (17). In fact, the levels of SETD2 and H3K36-me3 were lower in gILC than in IDC in our case. Because H3K36me3 is critical for recruiting the DNA mismatch repair (MMR) machinery, specifically the hMSH2–hMSH6 complex, to the chromatin, the loss of this histone mark results in a classical MMR-deficient phenotype characterized by MSI and an increased mutation rate (18). The high TMB observed exclusively in gILC may, in part, be associated with the *SETD2* mutation. Moreover, the uniquely identified mutational signatures SBS6 and SBS26 in gILC, both among the seven MMR–MSI-associated signatures (COSMIC, Mutational Signatures version 3.4, https://cancer.sanger.ac.uk/signatures/sbs/), further support the involvement of *SETD2*. ILC has higher TMB than IDC (19–21). Among the growth patterns of ILC, the solid pattern is significantly associated with a higher TMB (14). Fisher et al. reported that three of five glycogen-rich ILCs were confluent variety (2). In our case, the ILC component showed a solid pattern, which likely corresponded to the confluent type, suggesting that glycogen-rich clear ILC often has high TMB. A previous analysis of one MDL showed that the lobular component harbored more mutations and multiple unique driver gene alterations than the ductal component in a case of pleomorphic ILC (6). In metastatic ILC, high TMB is associated with T-cell-inflamed scores comparable to those with low TMB, suggesting the need for additional markers, such as programmed death-ligand, to predict the efficacy of immune checkpoint inhibitors (20). Given that MDL shows marked intercomponent heterogeneity in genetic alterations and gene expression (6–9), whether such molecular differences between components can guide treatment decisions warrants further investigation.

In conclusion, we reported a case of MDL predominantly exhibiting gILC coexisting with cILC and IDC. Genetic analysis suggests a common ancestor for gILC and IDC. The *SETD2* truncating mutation was identified only in the ILC component, likely contributing to the high TMB. HNF1B overexpression may be related to their glycogen-rich clear cell morphology. Further studies are required to clarify the underlying molecular mechanisms and their prognostic implications.

## Data Availability

All data generated or analyzed during this study are included in this published article/**Supplementary Material**.
