# Supplementary material for: Case Report: Mixed ductal–lobular carcinoma consisting of invasive lobular carcinoma with a glycogen-rich clear cell pattern and elevated tumor mutation burden
Source: Front Oncol. 2026 Jan 26;16:1741727. doi: 10.3389/fonc.2026.1741727 (PMC12884834; doi:10.3389/fonc.2026.1741727)
Supplement: Supplementary file 1 [file DataSheet1.docx]

**Supplementary Methods**

**Histopathological and immunohistochemical analyses**

Surgically resected tissue specimens were fixed in 10% buffered formalin, embedded in paraffin, and stained with hematoxylin and eosin (H&E). The histology and histological grade of these tumors were evaluated according to the fifth edition of the World Health Organization Classification of Tumours [1]. Immunohistochemical analyses were performed on sections from representative paraffin blocks containing each component. Heat-induced antigen retrieval was performed on each paraffin section. The following primary antibodies were used: estrogen receptor (ER) (SP1, pre-diluted, Roche, Basel, Switzerland), progesterone receptor (PgR) (1E2, pre-diluted, Roche), human epidermal growth factor receptor 2 (HER2) (4B5, pre-diluted, Roche), Ki-67 (MIB-1, pre-diluted, Agilent, Santa Clara, CA, USA), E-cadherin (NCH-38, 1:100, Agilent), HNF1-B (Rabbit polyclonal, 1:150, GeneTex, Irvine, CA, USA), SETD2 (E4W8Q, 1:250, IHC-Formulated, Cell Signaling Technology, Danvers, MA, USA), and tri-methyl-histone H3 (Lys36)(D5A7, 1:100, Cell Signaling Technology). For all markers except E-cadherin, HNF1-B, SETD2, and tri-methyl-histone H3, immunohistochemical procedures were performed using the automatic staining machine, VENTANA BenchMark ULTRA IHC/ISH system (Roche). ER, PgR, and HER2 were detected using the iVIEW DAB Detection Kit (Roche), while Ki-67 was detected using the ultraView Universal DAB Detection Kit (Roche). For E-cadherin, immunostaining was performed using HISTOSTAINER 48A (Nichirei, Tokyo, Japan), and E-cadherin expression was detected using the Histofine Simple Stain MAX-PO kit (Nichirei). Positive controls were used. ER, PgR, and HER2 were evaluated according to the American Society of Clinical Oncology/College of American Pathologists guidelines [2].

**Macrodissection**

Ten-micrometer-thick sections were prepared from representative formalin-fixed, paraffin-embedded (FFPE) blocks of tumor and normal breast tissues. Morphologically distinct components, namely, invasive lobular carcinoma with glycogen-rich clear cell morphology (gILC) and invasive ductal carcinoma (IDC), were macrodissected separately using a sterile needle. In addition, normal breast tissue was macrodissected and found to be devoid of neoplastic cells.

**Whole-exome sequencing**

Multiple thin sections were prepared in succession from each FFPE tissue block, and the first section was stained with H&E. Based on the results of H&E staining, tissues from the IDC or gILC components were manually obtained from the next 10 consecutive thinly sliced sections through macrodissection. DNA was extracted from the IDC or gILC components using the GeneRead DNA FFPE Kit (Qiagen, Hilden, Germany), in accordance with the manufacturer’s instructions to reduce artifactual C>T|G>A transitions. DNA was extracted from nonneoplastic breast tissue in each case using the same procedure to obtain the reference germline sequence. Double-stranded DNA was quantified using a Qubit 2 (Thermo Fisher Scientific, Waltham, MA). DNA purity was evaluated using a NanoPhotometer (Implen, Munich, Germany) as A260/A280 and A260/230 optical density ratios. Whole-exome sequencing (WES) was performed by Takara Bio Inc. (Shiga, Japan) for primary data analysis. Briefly, sequencing libraries were constructed from 100 ng of extracted DNA using the SureSelect XT HS DNA Reagent Kit and Human All Exon V6 (Agilent Technologies) and sequenced on an Illumina Hiseq2500.

**Analysis of single-nucleotide variants and short insertions and deletions (indels)**

We used FastQ (https://www.bioinformatics.babraham.ac.uk/projects/fastqc/) to confirm the read quality. The obtained sequence data were analyzed using the Genomon 2 DNA Analysis Pipeline (https://github.com/Genomon-Project) at the Human Genome Center, Institute of Medical Science, University of Tokyo (Tokyo, Japan). Single-nucleotide variants (SNVs) with minimum depth ≥ 8, base quality ≥ 15, variant read ≥ 4, P < 0.01 (Fisher’s exact test), and variant allele frequency ≥ 0.02 in the tumor were first selected for analysis. SNVs in the protein coding regions that resulted in changes in amino acid sequences were further evaluated to identify pathogenic or likely pathogenic variants by interrogating against the Catalogue of Somatic Mutations in Cancer (COSMIC) (https://cancer.sanger.ac.uk/cosmic), 1000 Genomes Project (https://international genome.org), dbSNPs (NCBI, NIH; https://www.ncbi.nlm.nih.gov/snp/), Functional Analysis through Hidden Markov Models (version 2.3) (http://fathmm.biocompute.org.uk), ClinVar (NCBI, NIH; https://www.ncbi.nlm.nih.gov/clinvar/), and OncoKB (Memorial Sloan Kettering Cancer Center; https://www.oncokb.org/) databases. A literature search was conducted to identify pathogenic and likely pathogenic SNVs.

**Analysis of COSMIC single-base substitution mutation signatures**

The R package “Mutational patterns 3.19.0” was used to analyze COSMIC SBS mutation signatures (human cancer signature version 3.4; https://cancer.sanger.ac.uk/signatures/sbs/). To reduce overfitting, an iterative procedure was implemented whereby, at each iteration, the signature with the lowest contribution was removed and the model was refitted. At each step, the cosine similarity between the original and the reconstructed profiles was calculated. The signature removal process was terminated when the difference in similarity between two successive iterations exceeded a predefined threshold. In this study, the threshold was set as *max_delta* = 0.004.

**Estimation of DNA copy number variants**

Copy number variant profiling was performed using DNAcopy version 1.56.0. (https://bioconductor.org/packages/release/bioc/html/DNAcopy.html) and the R/Bioconductor package.

**Phylogenetic tree construction**

MesKit version 1.6.0, an R package [3], was used to analyze the clonal evolution of invasive lobular carcinoma with glycogen-rich clear cell morphology and invasive ductal carcinoma based on the WES datasets.

**RNA-sequencing and fusion transcript identification**

Total RNA was isolated from FFPE samples using NucleoSpin RNA XP (MACHEREY-NAGEL GmbH & Co. KG, Duren, Germany), according to the manufacturer’s instructions. Thereafter, RNA purity was evaluated based on the optical density ratios (A260/A280 and A260/A230) determined using a NanoPhotometer (Implen, Munich, Germany). To assess RNA integrity, the RNA Nano 6000 Assay Kit and Agilent Bioanalyzer 2100 system (Agilent Technologies) were used. All RNA samples obtained were subjected to quality inspection, followed by library construction using TruSeq RNA Access (Illumina). This was followed by sequencing using an Illumina Hiseq2500 system.

**Gene expression profiling and pathway analysis**

We used FastQ (https://www.bioinformatics.babraham.ac.uk/projects/fastqc/) to confirm the read quality. Next, alignment of the human genome GRCh37 using STAR-2.5.2a (https://github.com/alexdobin/STAR/releases/tag/2.5.2a) and quality control analysis of the binary sequence alignment map for each sample were performed using the Genomon2 RNA analysis pipeline (https://github.com/Genomon-Project). The mapped read count for each sample was calculated using HTseq (https://htseq.readthedocs.io/en/release_0.9.1/). The differentially expressed genes (DEGs) were identified between two groups from the read count data using “DESeq2” Bioconductor R package (version 1.26.1) (https://bioconductor.org/packages/devel/bioc/vignettes/DESeq2/inst/doc/DESeq2.html). The threshold value to list DEGs showed a greater than twofold change, with p-value < 0.05 or adjusted p-value (p.adjust) < 0.05. Enrichment analysis was performed using the Metascape software (version 3.5) (<http://metascape.org>) [4]. Data were analyzed using Ingenuity Pathway Analysis (IPA) (QIAGEN IPA software, version 01-22-01, QIAGEN Inc., <https://www.qiagenbioinformatics.com/products/ingenuity-pathway-analysis>) for canonical pathway analyses.

**References**

[1] WHO Classification of Tumours Editorial Board. Breast tumours. Lyon (France): International Agency for Research on Cancer; 2019. (WHO classification of tumours series, 5th ed.; vol. 2). https://publications.iarc.fr/581.

[2] Allison KH, Hammond MEH, Dowsett M, McKernin SE, Carey LA, Fitzgibbons PL, et al. Estrogen and progesterone receptor testing in breast cancer: ASCO/CAP guideline update. J Clin Oncol. 2020;38:1346–66. <https://doi.org/10.1200/JCO.19.02309>

[3] Liu M, Chen J, Wang X, Wang C, Zhang X, Xie Y, et al. MesKit: A tool kit for dissecting cancer evolution of multi-region tumor biopsies through somatic alterations. GigaScience. 2021;10:giab036. <https://doi.org/10.1093/gigascience/giab036>

[4] Zhou Y, Zhou B, Pache L, Chang M, Khodabakhshi AH, Tanaseichuk O, et al. Metascape provides a biologist-oriented resource for the analysis of systems-level datasets. Nat Commun. 2019;10:1523. <https://doi.org/10.1038/s41467-019-09234-6>
