# Supplementary material for: Case Report: Mixed ductal–lobular carcinoma consisting of invasive lobular carcinoma with a glycogen-rich clear cell pattern and elevated tumor mutation burden
Source: Front Oncol. 2026 Jan 26;16:1741727. doi: 10.3389/fonc.2026.1741727 (PMC12884834; doi:10.3389/fonc.2026.1741727)
Supplement: Supplementary file 2 [file Image1.pdf]

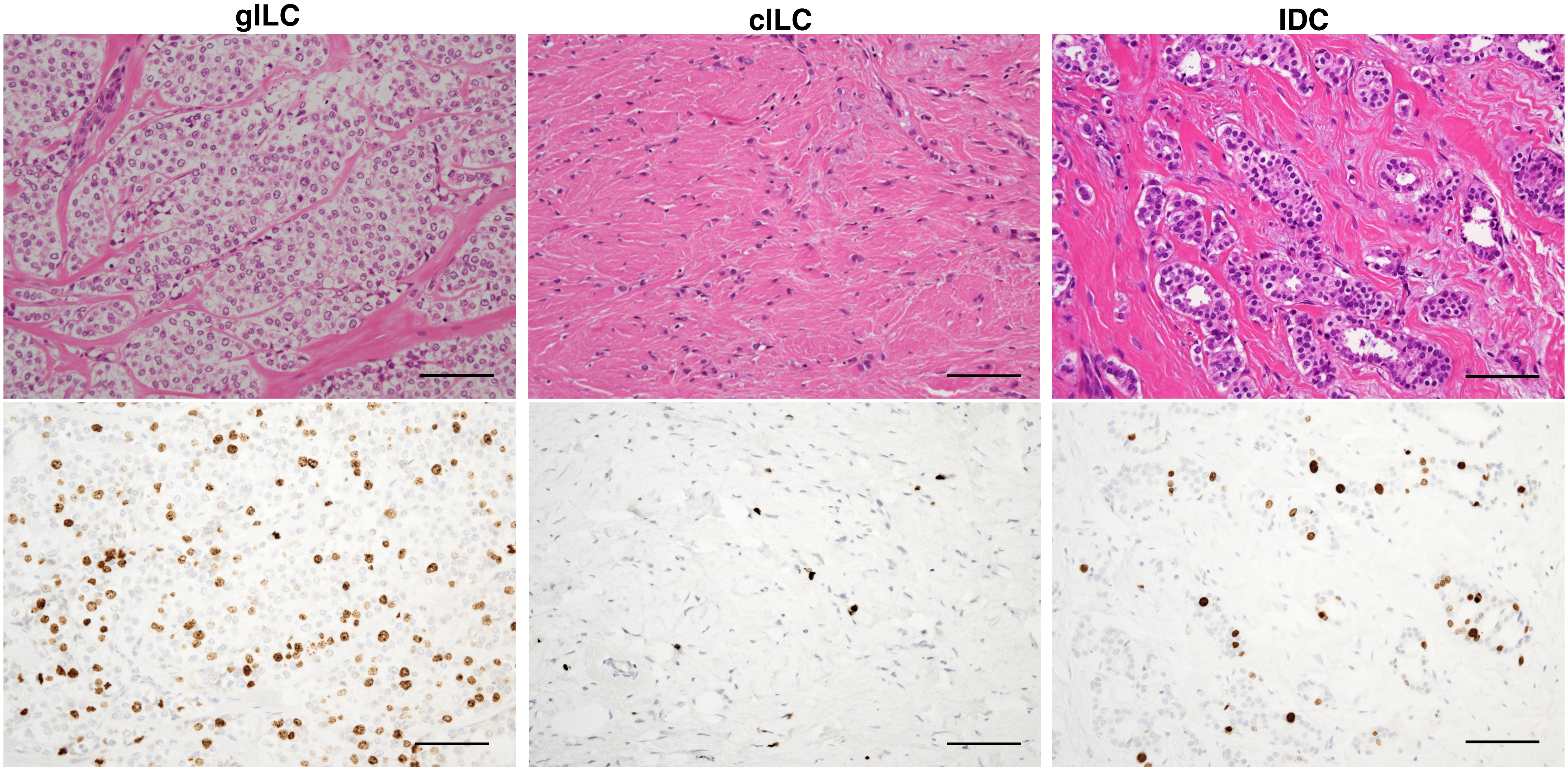

Supplementary Figure S1 Ki-67 proliferative indices. The Ki-67 positivity rate (hot spot) was 30% in gILC, 6% in cILC, and 10% in IDC, as evaluated by immunohistochemistry. Scale bar = 0.1 mm. gILC, invasive lobular carcinoma with a glycogen-rich clear cell pattern; cILC, classic invasive lobular carcinoma without a glycogen-rich clear cell pattern; IDC, invasive ductal carcinoma.
