# Supplementary material for: Case Report: Mixed ductal–lobular carcinoma consisting of invasive lobular carcinoma with a glycogen-rich clear cell pattern and elevated tumor mutation burden
Source: Front Oncol. 2026 Jan 26;16:1741727. doi: 10.3389/fonc.2026.1741727 (PMC12884834; doi:10.3389/fonc.2026.1741727)
Supplement: Supplementary file 3 [file Image2.pdf]

A

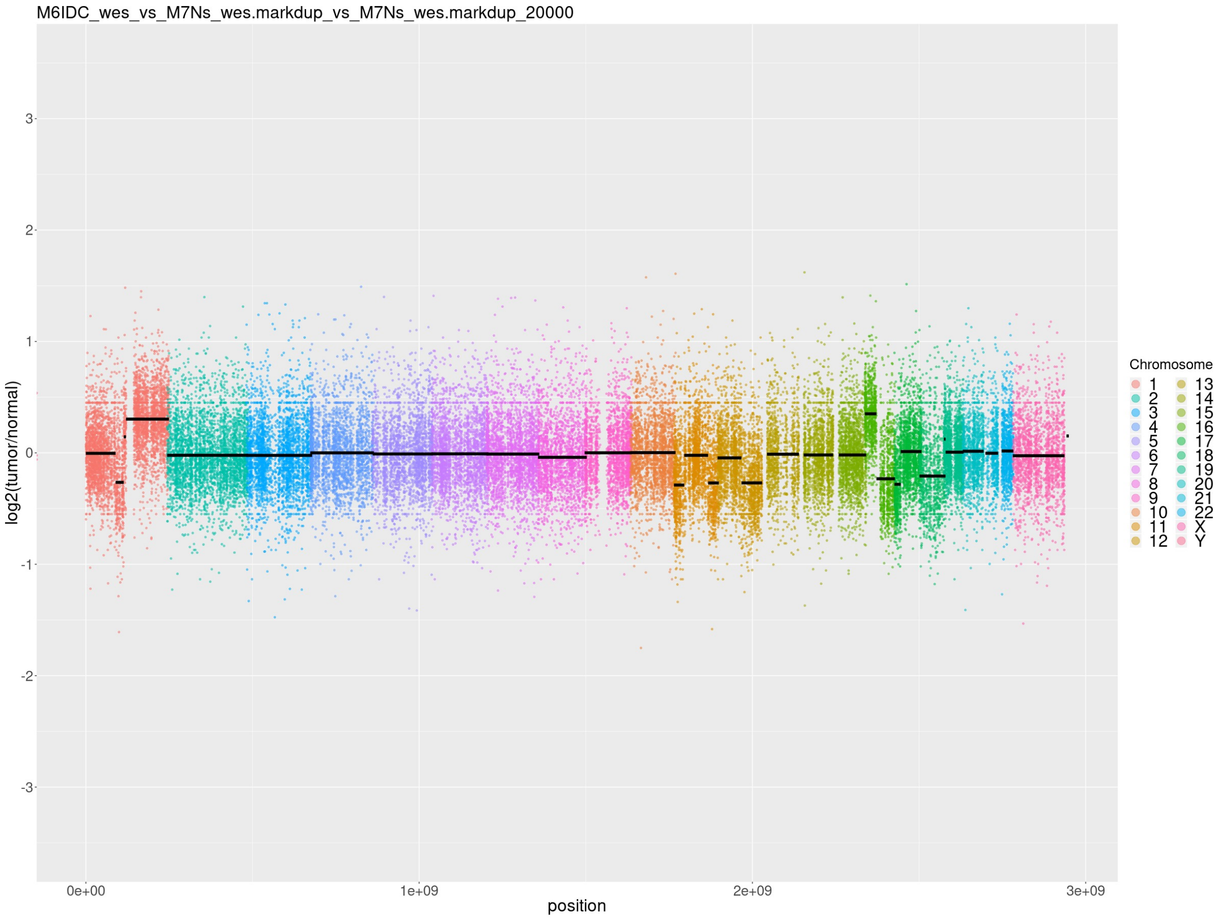
M6IDC_wes_vs_M7Ns_wes.markdup_vs_M7Ns_wes.markdup_20000
log2(tumor/normal)
position
Chromosome
1
2
3
4
5
6
7
8
9
10
11
12
13
14
15
16
17
18
19
20
21
22
X
Y

B

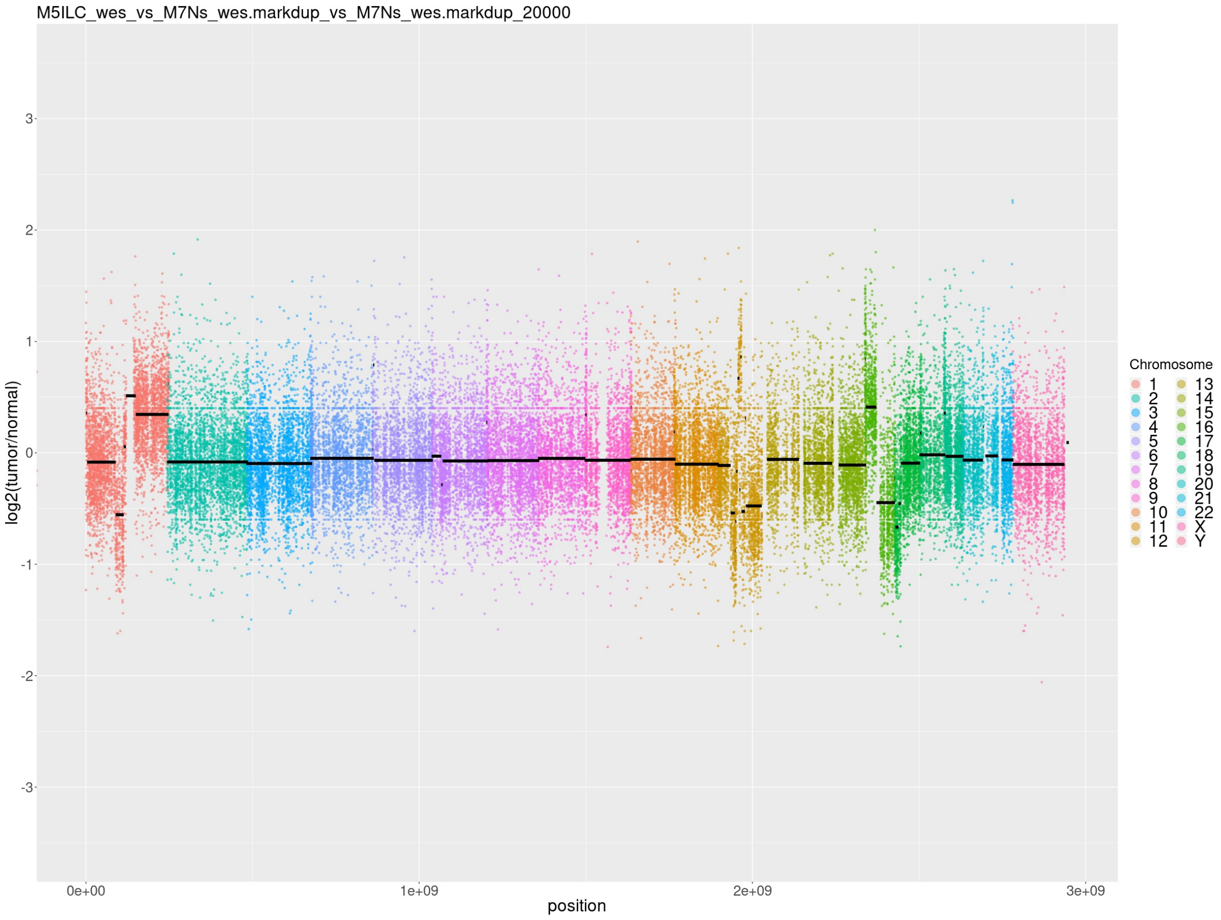
M5ILC_wes_vs_M7Ns_wes.markdup_vs_M7Ns_wes.markdup_20000
log2(tumor/normal)
position
Chromosome
1
2
3
4
5
6
7
8
9
10
11
12
13
14
15
16
17
18
19
20
21
22
X
Y

Supplementary Figure S2 Copy number analysis using whole-exome sequencing data from (A) IDC and (B) gILC. The y-axis represents the log2 copy number ratio of tumor versus normal tissue, and the x-axis represents the chromosomal position. Each dot corresponds to the copy number ratio of a genomic region and is colored by chromosome. The black lines indicate the segmented mean copy number levels smoothed by the R package DNAcopy. The profiles of IDC and gILC are similar, showing no significant copy number amplifications or deletions across the genome. IDC, invasive ductal carcinoma; gILC, invasive lobular carcinoma with glycogen-rich clear cell pattern.
