# Supplementary material for: Case Report: Mixed ductal–lobular carcinoma consisting of invasive lobular carcinoma with a glycogen-rich clear cell pattern and elevated tumor mutation burden
Source: Front Oncol. 2026 Jan 26;16:1741727. doi: 10.3389/fonc.2026.1741727 (PMC12884834; doi:10.3389/fonc.2026.1741727)
Supplement: Supplementary file 4 [file Image3.pdf]

A

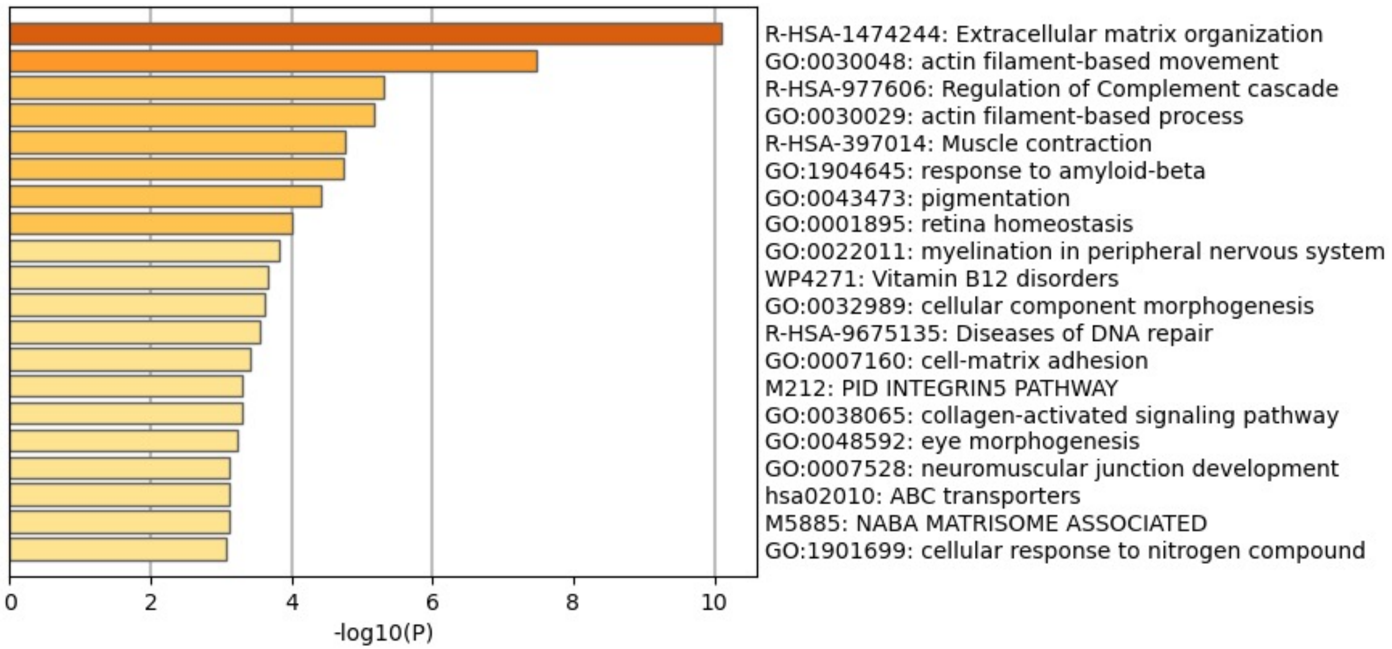
R-HSA-1474244: Extracellular matrix organization
GO:0030048: actin filament-based movement
R-HSA-977606: Regulation of Complement cascade
GO:0030029: actin filament-based process
R-HSA-397014: Muscle contraction
GO:1904645: response to amyloid-beta
GO:0043473: pigmentation
GO:0001895: retina homeostasis
GO:0022011: myelination in peripheral nervous system
WP4271: Vitamin B12 disorders
GO:0032989: cellular component morphogenesis
R-HSA-9675135: Diseases of DNA repair
GO:0007160: cell-matrix adhesion
M212: PID INTEGRIN5 PATHWAY
GO:0038065: collagen-activated signaling pathway
GO:0048592: eye morphogenesis
GO:0007528: neuromuscular junction development
hsa02010: ABC transporters
M5885: NABA MATRISOME ASSOCIATED
GO:1901699: cellular response to nitrogen compound
0
2
4
6
8
10
-log10(P)

B

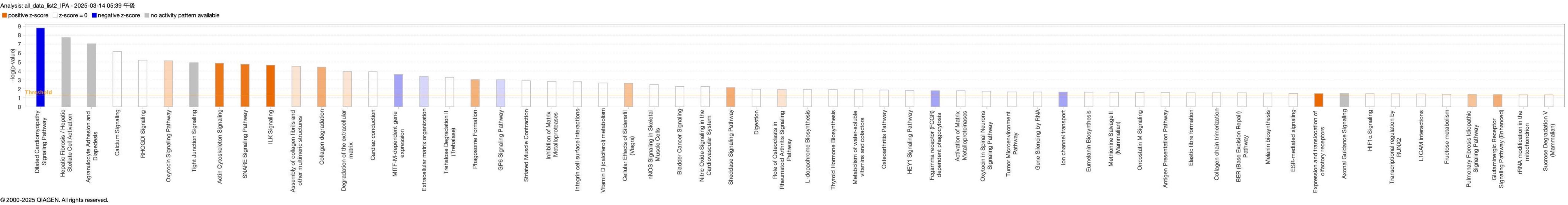
Analysis: all_data_list2_IPA - 2025-03-14 05:39 午後
positive z-score
z-score = 0
negative z-score
no activity pattern available
-log(p-value)
Threshold
Dilated Cardiomyopathy Signaling Pathway
Hepatic Fibrosis / Hepatic Stellate Cell Activation
Agranulocyte Adhesion and Diapedesis
Calcium Signaling
RHOGDI Signaling
Oxytocin Signaling Pathway
Tight Junction Signaling
Actin Cytoskeleton Signaling
SNARE Signaling Pathway
ILK Signaling
Assembly of collagen fibrils and other multimeric structures
Collagen degradation
Degradation of the extracellular matrix
Cardiac conduction
MITF-M-dependent gene expression
Extracellular matrix organization
Trehalose Degradation II (Trehalase)
Phagosome Formation
GP6 Signaling Pathway
Striated Muscle Contraction
Inhibition of Matrix Metalloproteases
Integrin cell surface interactions
Vitamin D (calciferol) metabolism
Cellular Effects of Sildenafil (Viagra)
nNOS Signaling in Skeletal Muscle Cells
Bladder Cancer Signaling
Nitric Oxide Signaling in the Cardiovascular System
Sheddase Signaling Pathway
Digestion
Role of Osteoclasts in Rheumatoid Arthritis Signaling Pathway
L-dopachrome Biosynthesis
Thyroid Hormone Biosynthesis
Metabolism of water-soluble vitamins and cofactors
Osteoarthritis Pathway
HEY1 Signaling Pathway
Fcgamma receptor (FCGR) dependent phagocytosis
Activation of Matrix Metalloproteinases
Oxytocin in Spinal Neurons Signaling Pathway
Tumor Microenvironment Pathway
Gene Silencing by RNA
Ion channel transport
Eumelanin Biosynthesis
Methionine Salvage II (Mammalian)
Oncostatin M Signaling
Antigen Presentation Pathway
Elastic fibre formation
Collagen chain trimerization
BER (Base Excision Repair) Pathway
Melanin biosynthesis
ESR-mediated signaling
Expression and translocation of olfactory receptors
Axonal Guidance Signaling
HIF1α Signaling
Transcriptional regulation by RUNX2
L1CAM interactions
Fructose metabolism
Pulmonary Fibrosis Idiopathic Signaling Pathway
Glutaminergic Receptor Signaling Pathway (Enhanced)
rRNA modification in the mitochondrion
Sucrose Degradation V (Mammalian)
© 2000-2025 QIAGEN. All rights reserved.

Supplementary Figure S3 Enrichment analysis of differentially expressed genes between gILC and IDC. (A) Enrichment analysis performed using Metascape. The bar plot shows the top enriched processes and pathways ranked based on statistical significance. **(B)** Canonical pathway analysis of differentially expressed genes between gILC and IDC using Ingenuity Pathway Analysis. The bar graph show clusters with representative enriched terms (one per cluster). The height of each bar represents the significance of the enrichment. The orange bars indicate predicted pathway activation (Z-score ≥ 2.0), blue bars indicate predicted pathway inhibition (Z-score ≤ –2.0) in IDC compared with gILC, and gray bars represent pathways where no prediction could be made or with Z-scores between –2.0 and 2.0. The orange line denotes the threshold for significance ($p < 0.05$). IDC, invasive ductal carcinoma; gILC, invasive lobular carcinoma with glycogen-rich clear cell pattern.
